# Structures of MERS1, the 5′ processing enzyme of mitochondrial mRNAs in *Trypanosoma brucei*

**DOI:** 10.1261/rna.072231.119

**Published:** 2020-01

**Authors:** Maria A. Schumacher, Max Henderson, Wenjie Zeng

**Affiliations:** Department of Biochemistry, Duke University School of Medicine, Durham, North Carolina 27710, USA

**Keywords:** MERS1, Nudix hydrolase, mRNA processing, kinetoplastid, *Trypanosoma brucei*

## Abstract

Most mitochondrial mRNAs are transcribed as polycistronic precursors that are cleaved by endonucleases to produce mature mRNA transcripts. However, recent studies have shown that mitochondrial transcripts in the kinetoplastid protozoan, *Trypanosoma brucei*, are transcribed individually. Also unlike most mitochondrial mRNAs, the 5′ end of these transcripts harbor a triphosphate that is hydrolyzed. This modification is carried out by a putative Nudix hydrolase called MERS1. The Nudix motif in MERS1 is degenerate, lacking a conserved glutamic acid, thus it is unclear how it may bind its substrates and whether it contains a Nudix fold. To obtain insight into this unusual hydrolase, we determined structures of apo, GTP-bound and RNA-bound *T. brucei* MERS1 to 2.30 Å, 2.45 Å, and 2.60 Å, respectively. The MERS1 structure has a unique fold that indeed contains a Nudix motif. The nucleotide bound structures combined with binding studies reveal that MERS1 shows preference for RNA sequences with a central guanine repeat which it binds in a single-stranded conformation. The apo MERS1 structure indicates that a significant portion of its nucleotide binding site folds upon substrate binding. Finally, a potential interaction region for a binding partner, MERS2, that activates MERS1 was identified. The MERS2-like peptide inserts a glutamate near the missing Nudix acidic residue in the RNA binding pocket, suggesting how the enzyme may be activated. Thus, the combined studies reveal insight into the structure and enzyme properties of MERS1 and its substrate-binding activities.

## INTRODUCTION

Kinetoplastids, a group of flagellated protists that include the parasitic protozoa, *Trypanosoma brucei*, *T. cruzi*, and *Leishmania* spp., are named after their unusual mitochondrial DNA called the kinetoplast (Vickerman 1985; Stevens 2008). Kinetoplastid mitochondrial DNA is composed of a complex assembly of maxi-circle and mini-circle DNA. The kinetoplastid mitochondria is also the site of several biological processes unique to these protists, such as kinetoplastid RNA editing (kRNA editing) (Simpson et al. 2003; Aphasizhev and Aphasizheva 2011; Aphasizheva and Aphasizhev 2016; Read et al. 2016). Despite its unusual nature, the kinetoplastid mitochondrial DNA, like most eukaryotic mitochondrial DNA, encodes several key proteins (a total of 18 mRNA transcripts) that are part of the oxidative phosphorylation cycle as well as multiple rRNAs. These transcripts are encoded in the maxi-circle DNA while the mini-circles encode guide RNAs (gRNAs) that facilitate kRNA editing.

Aside from plants, eukaryotic mitochondrial transcripts are generally produced as polycistronic units that are cleaved internally to generate individual transcripts. In these cases, the mRNA transcripts are bordered by tRNAs and excised via RNase P and tRNase Z enzymes (Ojala et al. 1981; Former et al. 2007; Hartmann et al. 2009). Although kinetoplastid mitochondrial mRNAs are not flanked by tRNAs, it was thought that these transcripts were produced by a similar route as animal and fungal mitochondrial mRNAs. However, recent work in the Aphasizhev laboratory revealed the finding that these transcripts in *T. brucei* are, in fact, individually synthesized and subsequently processed at their 5′ and 3′ ends to form mature mRNAs (Sement et al. 2018; Mesitov et al. 2019). The 5′ ends are processed by a pyrophosphohydrolase complex termed the PPsome, which is composed of the so-called mitochondrial edited mRNA stability factors 1–3 (MERS1–MERS3). MERS1 is a 395 residue protein, MERS2, a 946 residue peptatricopeptide repeat polypeptide and MERS3, a 189 amino acid protein with no known motifs (Sement et al. 2018). These three proteins also interface with kRNA editing and RNA processing complexes including the RNA-editing substrate-binding complex (RESC) and the kinteoplastid polyadenylation complex (KPAC) (Aphasizhev and Aphasizheva 2011; Ammerman et al. 2013; Aphasizheva et al. 2014; Suematsu et al. 2016; Sement et al. 2018; Mesitov et al. 2019). MERS1 was shown to be the factor responsible for catalyzing the 5′ processing event while MERS2 and MERS3 activates its catalysis (Sement et al. 2018).

Consistent with its role as an mRNA processing activity, studies have shown that MERS1 knockdown affected all mitochondrial mRNAs, not just those that are edited (Hashimi et al. 2009). Overall, MERS1 shows no sequence homology with any previously structurally characterized protein. However, it does harbor a putative Nudix box, which is a signature sequence found in a large family of hydrolases (Mildvan et al. 2005; Lin et al. 2008; Srouji et al. 2017). Nudix hydrolases, which are widely distributed from bacteria to humans, are named for their conserved overall function, which is the hydrolysis of nucleoside diphosphates linked to other moieties, X (Mildvan et al. 2005; McLennan 2006; Kraszewska 2008; Lin et al. 2008; Hong et al. 2014; Hill et al. 2017; Srouji et al. 2017). The Nudix box is a 23 residue signature sequence, GX_5_EX_7_REUXEEXGU (where X is any residue and U corresponds to hydrophobic residues, usually Ile, Leu, or Val) that forms a loop–helix–loop motif (Mildvan et al. 2005; McLennan 2006). The conserved glutamic acid residues in the motif, REUXEE, bind metal ions, typically magnesium, and are key for catalysis. The Nudix box is embedded in a Nudix fold, which consists of α−β−α sandwich and is composed of ∼120 residues (Mildvan et al. 2005).

Interestingly, the MERS1 Nudix motif (KWTLLYERYKEAAIRTLWEETGI) lacks one of the key metal ion coordinating acidic residues, which in MERS1 is a threonine instead of an acidic residue. Metal coordination by the conserved acidic residues in the Nudix motif is required for catalysis by most Nudix enzymes. However, the mechanisms used by Nudix hydrolases are quite diverse (Mildvan et al. 2005). Specifically, while all involve hydrolysis, they differ in the position on the substrate where the nucleophilic substitution occurs. Moreover, while most Nudix hydrolases proceed by an associative nucleophilic substitution involving a water attacking an internal phosphorous of a diphosphate chain, members of the GDP-mannose hydrolase family catalyze dissociative nucleophilic reactions via water at a carbon (Legler et al. 2000; Gabelli et al. 2004; Mildvan et al. 2005; Lin et al. 2008; Srouji et al. 2017). Also, while the acidic residues within the Nudix motif are important for ion coordination, the number of bound metal ions vary (Srouji et al. 2017). Thus, there is significant catalytic versatility among Nudix hydrolases, which also makes it difficult to predict the substrate and catalytic mechanism of a given protein with a Nudix motif.

The presence of a nonconsensus Nudix motif in MERS1 raised the possibility that it may not contain a Nudix fold. Moreover, outside its putative Nudix motif, the protein shows no sequence homology with any structurally characterized protein. Studies support that MERS1 binds G-rich RNA sites; crosslinking and immunoprecipitation (CLIP) and crosslinking and affinity purification (CLAP) experiments uncovered a consensus motif of 5′-GAGAGGGGGGUUU-3′ that was associated with MERS1 and MERS2 (Sement et al. 2018). However, direct RNA binding by MERS1 has not been assessed. Thus, to gain insight into the structure and function of this hydrolase we determined crystal structures of MERS1 in its apo, GTP-bound and RNA-bound states and performed biochemical studies to probe substrate binding.

## RESULTS AND DISCUSSION

### Structure of the *T. brucei* MERS1 protein

**FIGURE 1.**
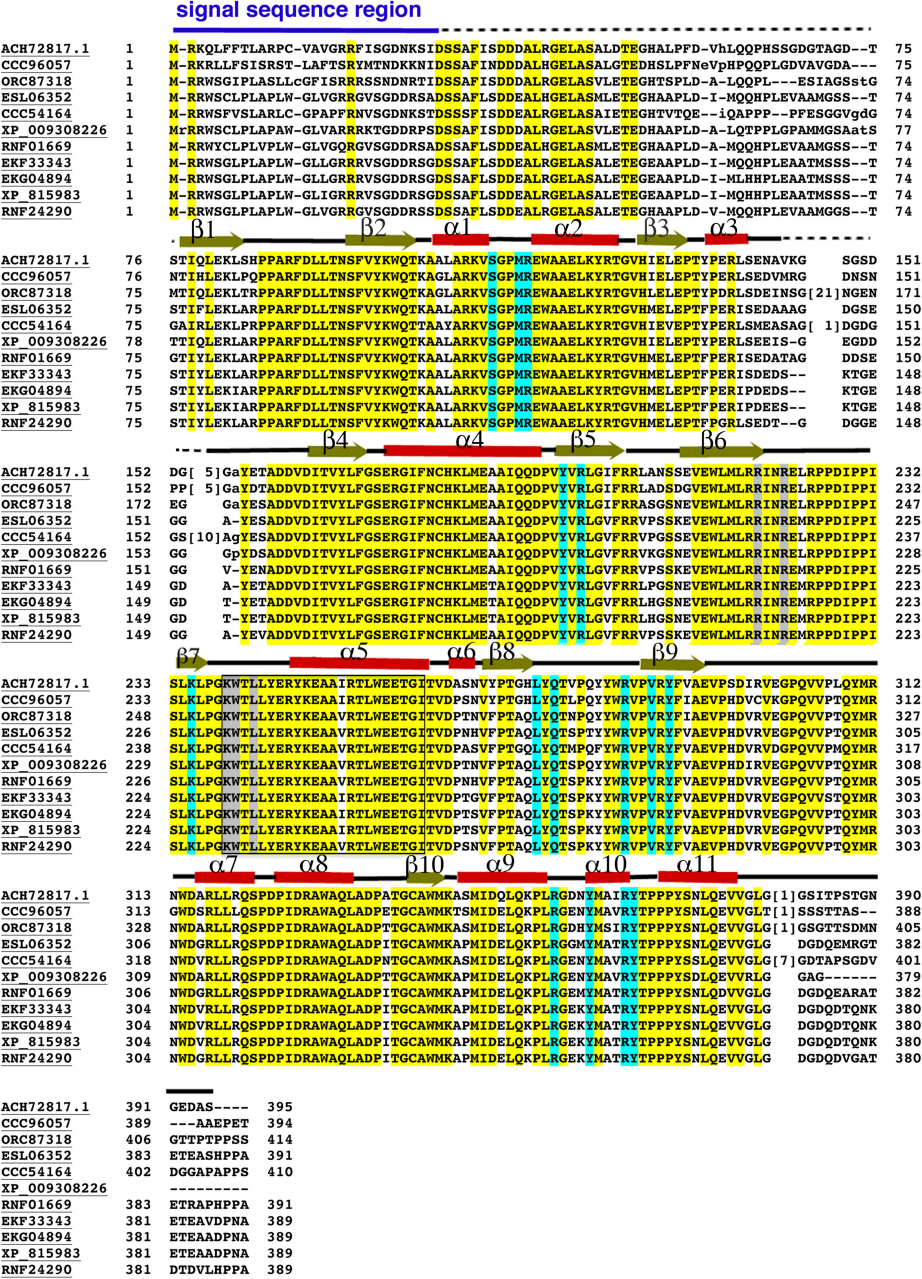
MERS1 sequence alignments. Sequence alignments of MERS1 proteins. The organism from which the MERS1 is derived is indicated by the code as follows. ACH72817.1, *Trypanosoma brucei brucei* TREU927 (the protein under study); CCC96057, *Trypanosoma congolense* IL3000; ORC87318, *Trypanosoma theileri*; ESL06352, *Trypanosoma rangeli* SC58; CCC54164, *Trypanosoma vivax* Y486; XP_009308226, *Trypanosoma grayi*: RNF01669, *Trypanosoma rangeli*; EKF33343, *Trypanosoma cruzi marinkellei*; EKG04894, *Trypanosoma cruzi*; XP_815983, *Trypanosoma cruzi* strain CL Brener; RNF24290, *Trypanosoma conorhini*. All yellow highlighted residues are invariant among homologs. Residues highlighted in gray contact the helix bound near the Nudix motif in the MERS1–GTP structure. Residues in cyan make hydrogen bonding or hydrophobic contacts to the nucleotides in the MERS1–RNA structure. Secondary structural elements of MERS1 are indicated *over* the sequence alignments. The Nudix motif is boxed, the putative signal sequence at the amino terminus that was not included in the construct is indicated by a blue line *over* the sequence and regions that were disordered or not resolved in the structure are denoted by a dashed black line *over* the sequence.

Multiple sequence alignments of MERS1 homologs revealed that the amino-terminal ∼70 residues of these proteins are poorly conserved. This is in contrast to the remainder of the protein sequence, which shows strong conservation among homologs (Fig. 1). Data analyses suggest that the amino-terminal region likely functions as a signal sequence; MERS1 is chromosomally encoded and the protein transported into the mitochondria. As signal sequences are typically not well ordered, we generated a truncation construct of the protein for structural studies. Because the extent of the signal sequence is not clear, we chose a conservative truncation, removing the first 36 residues. Crystals of the apo protein, MERS1–GTP complex and MERS1 bound to the RNA site 5′-GAGAGGGGGGUU-3′ were produced (Materials and Methods).

**FIGURE 2.**
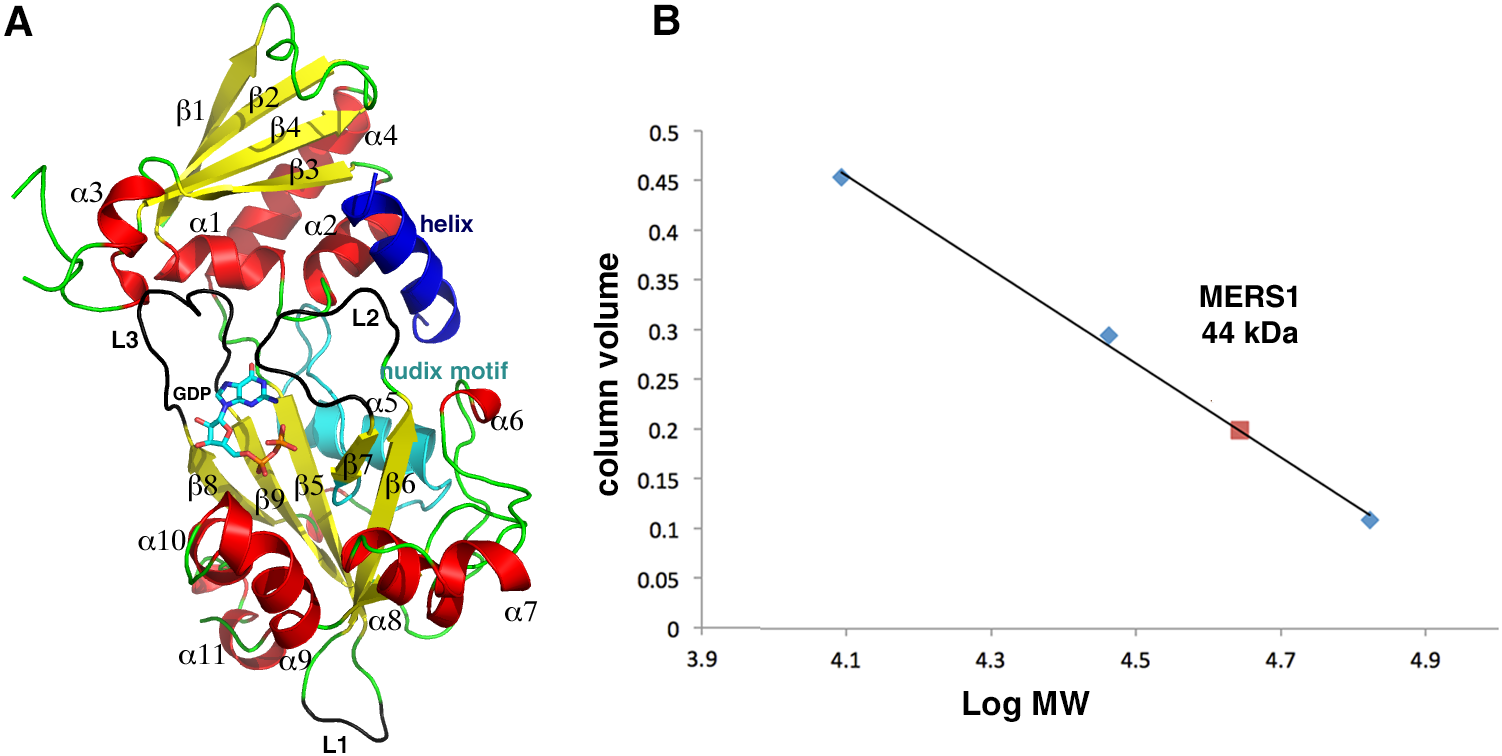
Crystal structure of *T. brucei* MERS1–GDP complex. (*A*) Ribbon diagram of the *T. brucei* MERS1–GDP complex. Secondary structural elements are labeled with helices colored red, strands, yellow and loops, green. The bound GDP is shown as sticks. The Nudix region is colored cyan and the helix bound from a symmetry mate in the crystal is colored blue. The loops that are unique to MERS1 are colored black and labeled L1, L2, and L3. All ribbon diagrams were made using PyMOL (Delano 2002). (*B*) Size exclusion chromatography analysis of the *T. brucei* MERS1. The *x* and *y* axes are Log MW and column volume, respectively. MERS1 eluted at a calculated MW of 44 kDa (red square), consistent with a monomer. The standards used for calculation of the standard curve are shown as blue diamonds and were cytochrome c oxidase (12.4 kDa), carbonic anhydrase (29 kDa), and albumin (66 kDa).

Crystals of the MERS1-GTP complex were obtained first and the structure was solved by multiple-wavelength anomalous diffraction (MAD) using a selenomethionine-substituted crystal (Materials and Methods) (Table 1). The structure was refined to final *R*_work_/*R*_free_ values of 22.9%/25.7% to 2.45 Å resolution (Table 2). The MERS1 structure reveals that it is comprised of two α−β domains (Fig. 2A). Domain 1 extends from residues 77 to 192. Domain 2 encompasses residue 193–377. Amino-terminal residues 37–76, carboxy-terminal residues 378–395, and a loop region from residues 145–155 are not visible in the structure. Notably, these disordered residues correspond to regions that are not conserved among MERS1 homologs (Figs. 1, 2A). A Nudix fold in the MERS1 structure encompasses residues 195–310 and within the Nudix fold is the Nudix motif, which like other Nudix hydrolase structures consists of a loop–helix–loop (Fig. 2A). The structure contains one MERS1 subunit in the crystallographic asymmetric unit (ASU) and no higher-order oligomers were revealed from crystallographic packing or PISA analyses, suggesting that the protein is monomeric (Krissinel and Hendrick 2007). However, to assess the oligomeric state of the protein in solution, we performed size exclusion chromatography (SEC) experiments (Materials and Methods). In these studies, MERS1(37–395) eluted from the superdex 75 (S75) gel filtration column at a molecular weight of 44 kDa, which is consistent with the calculated molecular weight for the his-tagged version of a MERS1(37–395) monomer of 42 kDa (Fig. 2B). Interestingly, clear electron density in the MERS1-GTP structure was observed for an extra helix docked between the two domains of MERS1, next to the Nudix motif (Fig. 2A). The presence of a selenomethionine site on the helix and the electron density identified the sequence as AAAGSMDDALRGELA (where the amino-terminal part of the peptide had weak density and hence was modeled as polyalanines). This sequence corresponds to the linkage between the end of the affinity tag (LVPRGSM) and the amino-terminal residues in the MERS1(37–395) construct (DDALRGELA). Analysis of the crystal packing indicated that this helix comes from a MERS1 molecule packed nearby in the crystal.

**TABLE 1.**
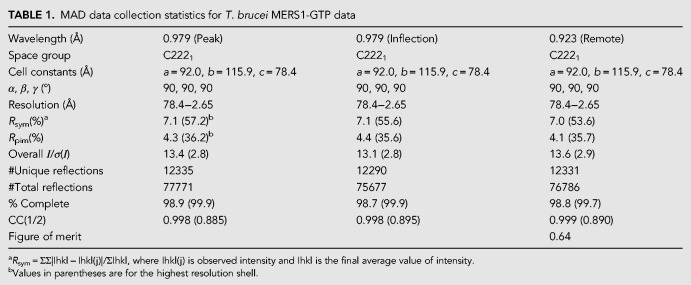
MAD data collection statistics for *T. brucei* MERS1-GTP data

**TABLE 2.**
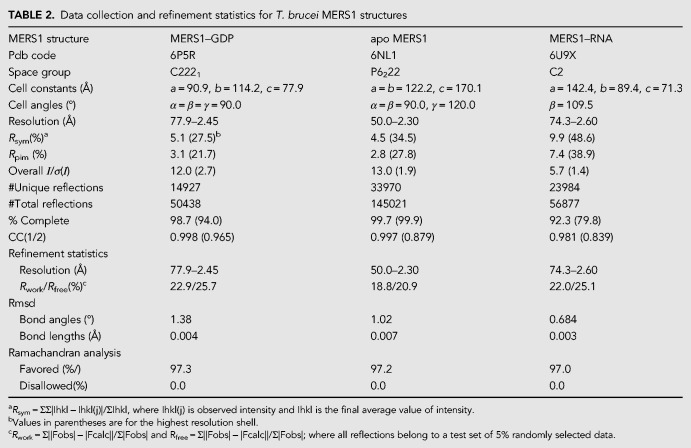
Data collection and refinement statistics for *T. brucei* MERS1 structures

Database (Dali) searches (Holm and Rosenstrom 2010) failed to identify structures that showed homology with the overall MERS1 fold, indicating it is unique. However, structures with similarities to the MERS1 Nudix fold were identified. Structures displaying the strongest similarity to that of MERS1 were the *Escherichia coli* Nudix hydrolase YmfB (Kraszewska 2008) and *E. coli* GDP-Mannose Manosyl hydrolase (Gabelli et al. 2004). These structures can be superimposed onto 109 and 122 Cα atoms of the MERS1 structure with root mean squared deviations (rmds) of 1.8 and 2.0 Å, respectively. The MERS1 structure, however, reveals that there are insertions within its Nudix fold compared to the other hydrolases, specifically, residues 207–210, 222–230, and 278–283 (labeled L1, L2, and L3 in Fig. 2A). Amino acids 207–210 form an extended loop far from the nucleotide-binding site. The region that encompasses residues 278–283 forms part of a shield over the guanine nucleotide-binding site and also interacts with domain 1 of MERS1. Residues 222–230 form an extended loop that also covers the nucleotide-binding pocket and interacts with the helix docked near the pocket (Fig. 2A).

### MERS1–GDP interactions

**FIGURE 3.**
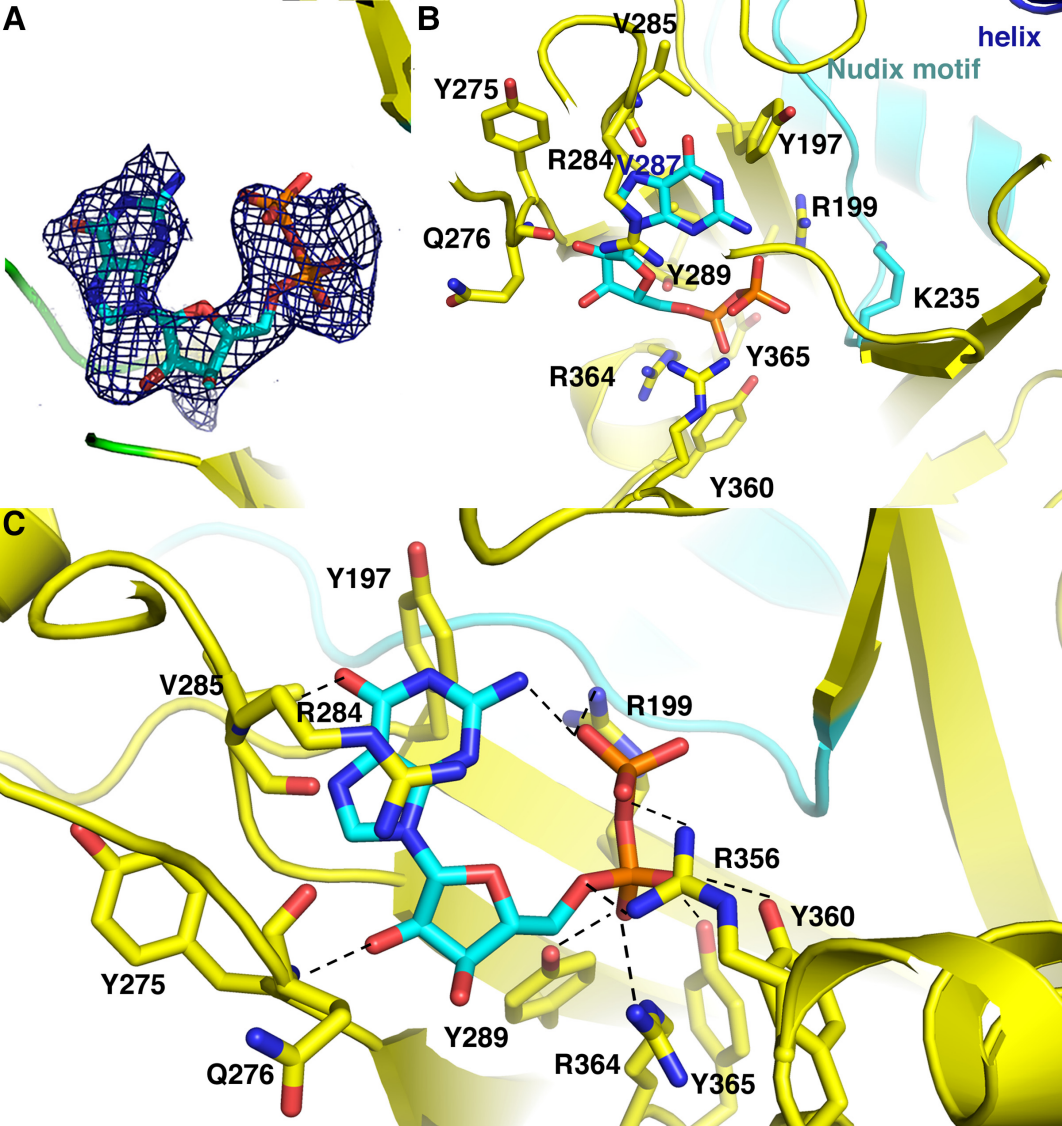
MERS1–GDP interactions. (*A*) Fo-Fc omit map (blue mesh), calculated before the addition of the nucleotide into the model and contoured at 3.4σ around the GDP molecule. (*B*) Close-up of MERS1–GDP interactions. Residues contacting the GDP are shown as sticks and labeled. The helix from packing interactions is shown in blue and labeled. (*C*) Close-up of the MERS1 interactions with GDP showing the residues that contact GDP.

Density for the nucleotide was evident in the MERS1–GTP structure, wedged into a pocket at the interface between the MERS1 domains. The GTP γ phosphate electron density is weak hence, we constructed the nucleotide as GDP (Fig. 3A). It is possible that the GTP may have been hydrolyzed or, more likely (see below), the missing density is due to the weak binding of the γ phosphate in the pocket. There are extensive interactions between MERS1 residues and the GDP in this binding site that effectively lock it in place. Lys235 from the Nudix motif makes electrostatic interactions with the GDP phosphate groups. A number of other MERS1 residues contact the GDP phosphate moieties including Arg199, Arg356, and Arg364 as well as tyrosine residues, Tyr289, Tyr360, and Tyr365 (Fig. 3B,C; Supplemental Fig. S1). Interactions to the ribose moiety are made by backbone atoms of residues Tyr275 and Gln276. Finally, there are numerous hydrophobic and stacking interactions from MERS1 residues to the guanine base. These are provided by the side chains of Tyr197, Tyr275, and Val285. In addition, the side chain of Arg284 makes cation-π interactions with the guanine base. These contacts shield the base from the solvent. The guanine base O6 atom makes a hydrogen bond to the amide nitrogen of MERS1 residue Val285 (Fig. 3C). Notably, these GDP interacting residues are conserved in MERS1 homologs (Fig. 1).

### MERS1-helical interaction

**FIGURE 4.**
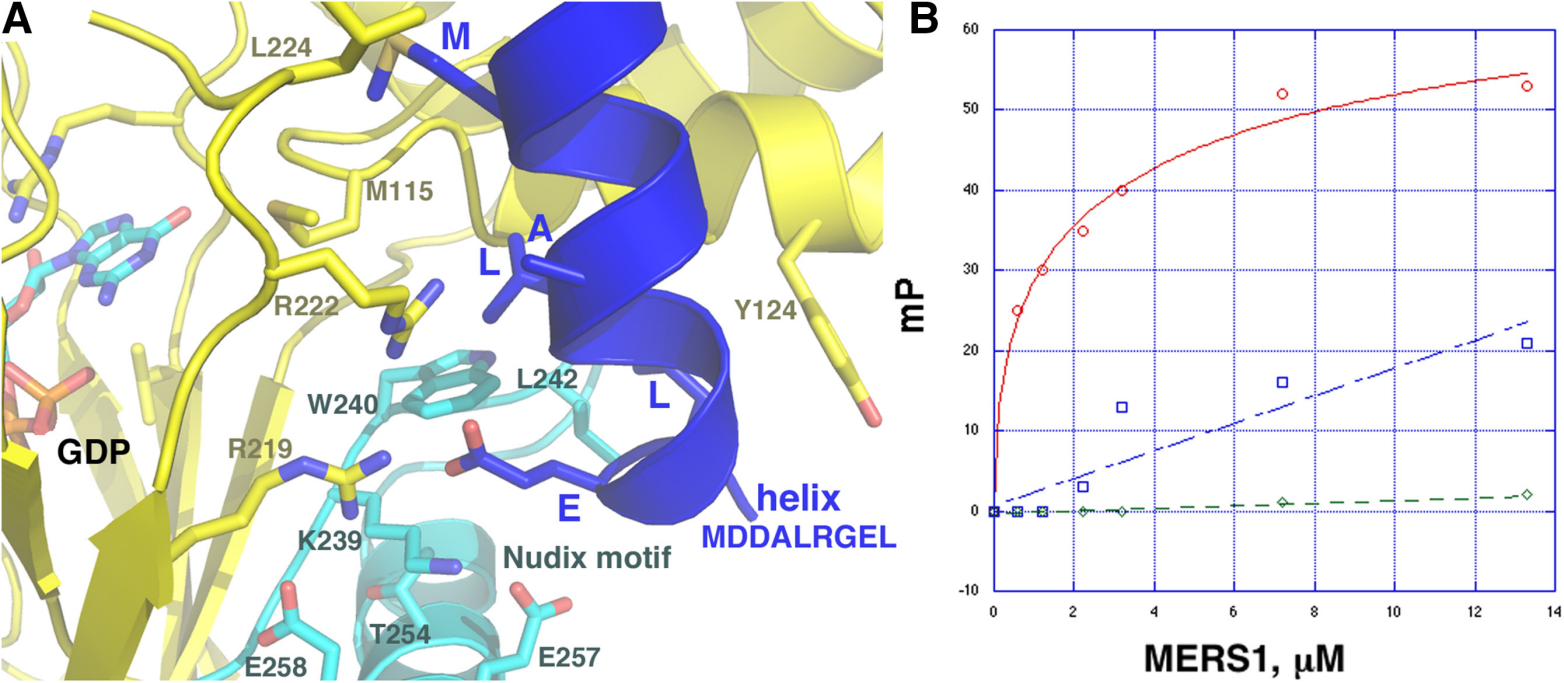
MERS1 binding to MERS2 peptide. (*A*) Close-up of the MERS1-(MERS2-like) helix interaction. The MERS1 residues that interact with the helix (blue) are shown as sticks and labeled. Residues in the Nudix motif are colored cyan. The residues that correspond to the acidic residues in other Nudix motifs (Thr254, Glu257, and Glu258) are shown as sticks and labeled. The figure highlights that the Nudix motif is close to the helix. (*B*) Fluorescence polarization (FP) binding analyses of fluoresceinated peptides binding to MERS1. The *y*-axis is in millipolarization units (mP) and the *x*-axis is MERS1 concentration (µM). The binding isotherm in red corresponds to that from the MER2 peptide, the blue isotherm is the same peptide but with proline mutations at residues within the helix that contact MERS1 (the proline substitutions are underlined; SVSKQAVCLMERHLRRVPGARRNELKVAPSKPLKER). The isotherm in green is the binding analyses of a non-MERS2 peptide. Each binding curve is a representative analysis from at least three technical replicates. The standard error was determined from the binding affinities of the three separate experiments.

Data indicate that MERS1 forms a complex with the MERS2 and MER3 proteins and that these interactions activate MERS1 activity (Sement et al. 2018). The 946 residue MERS2 protein contains several pentatricopeptide (PPR) motifs, which are interaction motifs that may be involved in binding MERS1 and/or RNA. PPR motifs are comprised of two helices that each contain three to four helical turns. We noted that the fortuitously bound helix in the MERS1 structure consists of three helical turns (Fig. 2A). This helix docks near the MERS1 Nudix motif and is proximal to the acidic residues of this motif. MERS1 makes a number of interactions with this helix, in particular with hydrophobic residues from the helix. For example, the helical leucine residues (XXXMDDALRGELA) make hydrophobic contacts to MERS1 Nudix motif residues Trp240 and Leu242 (Fig. 4A). The small size of the helix alanine side chain, amino-terminal to the first leucine, appears important in mediating the interaction as a large side chain could cause a clash with MERS1. The helix methionine residue also participates in hydrophobic contacts with MERS1. Finally, the helix glutamic acid contacts Lys239 from the Nudix motif as well as MERS1 residues Arg219 and Arg222 (Fig. 4A). It is notable that the helix glutamic acid is inserted very close to the positions, Thr254, Glu257, and Glu258, where acidic residues are found in canonical Nudix motifs.

We considered that the bound helix provided from crystal packing might mimic a portion of MERS2 or MERS3 that interacts with MERS1. Thus, using the MERS1-peptide contacts we derived a consensus MERS1-peptide interacting motif, (M/L)XXALXXEL and probed the MERS2 and MERS3 sequences for similar motifs. While no homology was found between the sequence and that of MERS3, one strong match for the motif was located within the central PPR motif region of MERS2 (residues 394–402). This region is predicted to be helical and contain a PPR motif in MERS2. Attempts to purify MERS2 or a MERS2 truncation containing this PPR motif for binding analyses, however, were unsuccessful. Thus, we obtained a fluoresceinated peptide encompassing this MERS2 region, F-SVSKQAVCLMERHLRRVPGARRNELKVALSKELKER (where underlined residues correspond to the peptide consensus residues) and utilized this peptide in FP binding studies. The experiments revealed that the peptide bound MERS1 saturably with a *K*_d_ of 2.5 ± 0.8 µM (Fig. 4B). This interaction appeared specific as MERS1 failed to interact with another fluoresceinated peptide that lacked the consensus residues, F-MSNNALDRLINKQKPKVPPRNDVVSESVSN (Fig. 4B). However, to test whether helical residues observed in the crystal to make contact to MERS1 are involved in binding, we obtained a fluoresceintated peptide in which the first leucine and the glutamic acid in the helix were substituted to prolines (F-SVSKQAVCLMERHLRRVPGARRNELKVAPSKPLKER). This peptide showed only weak binding to MERS1 (Fig. 4B). Because, as noted, the bound MERS2 helical peptide is close to the MERS1 Nudix motif, the interaction captured in the structure may suggest a possible mechanism for how MERS2 may activate MERS1.

### Apo MERS1 structure suggests nucleotide induced conformational change

**FIGURE 5.**
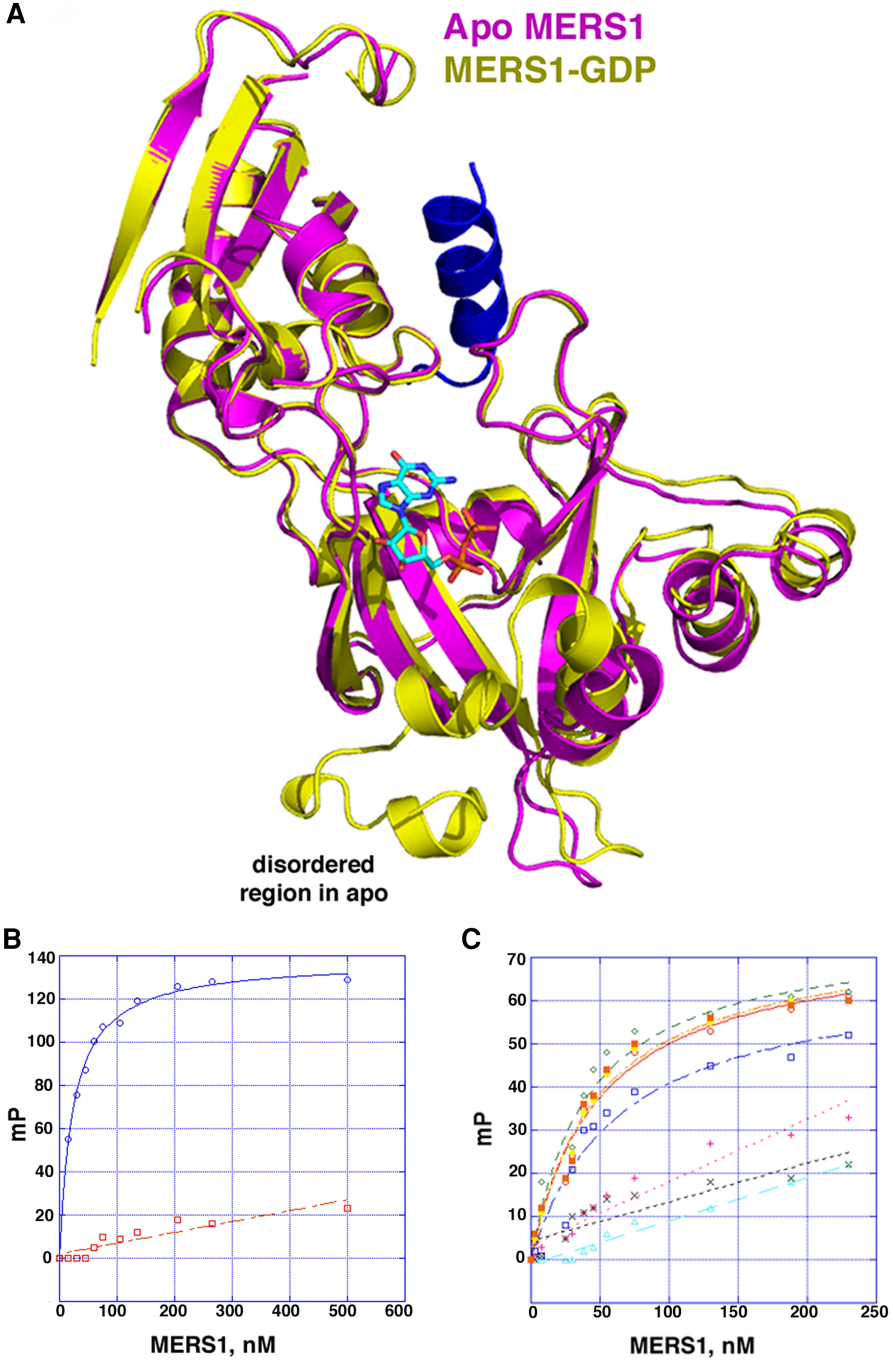
Nucleotide binding to MERS1: RNA-mediated folding and RNA binding specificity. (*A*) Superimposition of the apo MERS1 (magenta) and MERS1–GDP (yellow) structures. The helix bound in the MERS1–GDP structure is also shown (blue). The location of the bound GDP is also shown. The overlay shows that the carboxy-terminal region of the MERS1 structure that is involved in nucleotide binding is disordered in the apo structure. (*B*) FP binding isotherms investigating MERS1 binding to RNA and DNA. The RNA site, 5′-GAGAGGGGGUU-3′, binds MERS1 with high affinity (blue curve) and saturably while the DNA shows no binding (red curve). The *y*-axis is millipolarization units (mP) and the *x*-axis is the protein concentration in nM. Each binding curve is a representative analysis from at least three technical replicates. The standard error was determined from the binding affinities of the experiments. (*C*) RNA specificity FP binding studies. The titrations were performed as for *B*. Each binding curve represents a distinct RNA species in which single substitutions (underlined) were made in the binding site. The WT RNA, 5′-GAGAGGGGGUU-3′ (green diamonds), 5′-GGGAGGGGGUU-3′ (filled orange squares), 5′-GAAAGGGGGUU-3′ (blue unfilled squares), 5′-GAGGGGGGGUU-3′ (orange unfilled circles), 5′-GAGAAGGGGUU-3′ (yellow filled diamonds), 5′-GAGAGAGGGUU-3′ (magenta plus signs), 5′-GAGAGGAGGUU-3′ (gray crosses) and 5′-GAGAGGGAGUU-3′ (cyan circles). The resultant *K*_d_s are 40 ± 0.9 nM, 48 ± 0.9 nM, 78 ± 4 nM, 47 ± 5 nM, and 43 ± 5 nM. The last three substitutions (magenta, gray and cyan curves) did not bind saturably and so *K*_d_s were not determined. Each binding curve is a representative analysis from at least two technical replicates. The standard error was determined from the binding affinities of the experiments.

To ascertain any structural changes that may occur upon nucleotide binding in MERS1, we next determined the structure of the apo protein. Apo MERS1 crystals were grown and the structure was solved using the MERS1–GDP structure (after removing the GDP molecule) as a search model in molecular replacement (MR). A clear solution was obtained and the structure was refined to final *R*_work_/*R*_free_ values of 18.8%/20.9% to 2.30 Å resolution (Table 2). Like the MERS1–GDP structure, crystal packing analyses revealed no higher-order oligomeric structures, consistent with the protein existing as a monomer. The apo MERS1 adopts a different crystal form with distinct packing compared to the MERS1–GDP structure and as a result we did not see the fortuitously bound helix in the apo structure. Comparison of the apo and GDP bound MERS1 structure revealed that the N-domain and most of the C-domain have the same structure; the two structures can be superimposed with a rmsd of 0.9 Å for 260 corresponding Cα atoms. There is, however, a notable difference between the two structures at the base of C-domain near the GDP binding pocket. Specifically, in the apo structure density was absent for the region encompassing residues 354–380 (Fig. 5A). This region contains key residues involved in nucleotide binding, including Arg356, Tyr360, Arg364, and Tyr365. This disorder suggests that substrate binding is needed to construct the nucleotide-binding pocket.

### Characterization of RNA binding by MERS1

We next used FP binding studies to assess RNA binding by purified MERS1. These analyses showed that MERS1 bound the RNA, 5′-GAGAGGGGGGUU-3′, saturably with a *K*_d_ of 40 ± 0.9 nM (Materials and Methods). In contrast, a DNA site, 5′-GTGAGTACTCAC-3′, showed essentially no binding (Fig 5B). We next probed the importance of specific nucleotides in the RNA binding site by performing FP studies on mutant forms of the RNA site. The combined data showed that a guanine in the 5′ position of the binding site was not required for binding (Supplemental Fig. S2) and changing the adenine in the second position also had no effect (Fig. 5C). The nucleotides substitutions that resulted in reduced binding were in the third, sixth, seventh, and eighth positions in the RNA. Substitution of the third guanine to adenine resulted in a minor (approximately twofold) impairment in RNA binding. In contrast, the sixth, seventh, and eighth positions showed a preference for guanine, as their substitutions to adenine led to weak binding by MERS1 (Fig. 5C).

### MERS1–RNA structure

**FIGURE 6.**
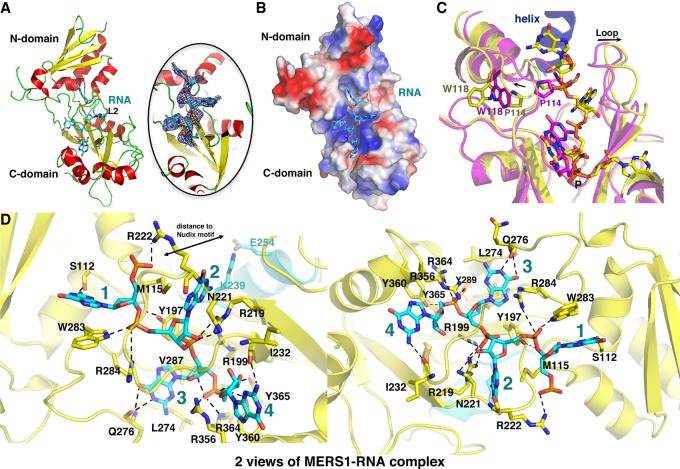
MERS1–RNA structure. (*A*) Overall structure of the MERS1-5′-GAGAGGGGGGUU-3′ complex. The MERS1 is shown as ribbons with strands, helices and loops colored yellow, red and green, respectively. The RNA is shown as cyan. *Right* is a close-up of the RNA binding site with an Fo-Fc map included that is contoured at 2.6σ around the RNA at 2.6 Å resolution. (*B*) Electrostatic surface representation of the MERS1–RNA structure where electropositive and electronegative regions are colored blue and red, respectively. Loop 2 (L2), which covers the RNA and shields it from solvent, was removed to visualize the individual RNA nucleotide-binding pockets. (*C*) Superimposition of the MERS1–GTP (magenta) structure onto the MERS1–RNA structure (yellow). The comparison shows that L2 and residues 111–128 shift upon RNA binding. Also shown is the location of the fortuitously bound helix (blue) in the MERS1-GTP structure, which is close to the RNA substrate in the MERS1–RNA complex. (*D*) MERS1–RNA contacts. Two views of the MERS1–RNA interaction are shown in different orientations. The nucleotides are numbered 1 to 4 in the 5′ to 3′ direction.

In the MERS1–GDP structure the GDP (GTP) is ∼15 Å from the Nudix motif and thus too far to be accessible for catalysis by residues in the Nudix motif, suggesting that it may not use a typical Nudix hydrolase type mechanism. Alternatively, the GTP might be bound at a site that does not correspond to the 5′ triphosphate binding pocket. Precedent for the latter possibility comes from studies on bacterial RNA pyrophosphohydrolase (RppH) enzymes (Gabelli et al. 2002; Messing et al. 2009; Hsieh et al. 2013; Piton et al. 2013; Vasilyev and Serganov 2015). RppH enzymes, like MERS1, carry out the conversion of 5′ triphosphate groups of their RNA substrates to 5′ monophosphorylated transcripts. Although all these enzymes catalyze the same mechanism, they show different binding preferences. The *Bdellovibrio bacteriovorus* RppH, for example, strongly prefers a guanosine in the 5′ position, while *Bacillus subtilis* and *Escherichia coli* proteins display a preference for binding guanine in the second position (Gabelli et al. 2002; Messing et al. 2009; Hsieh et al. 2013; Piton et al. 2013; Vasilyev and Serganov 2015). Also, the *E. coli* and *B. bacteriovorus* catalyze the conversion of RNA 5′ triphosphate to 5′ monophosphate in a single step, with pyrophosphate release, while *B. subtilis* RppH carries out this reaction in two steps (Deana et al. 2008; Messing et al. 2009; Piton et al. 2013). Furthermore, the *B. subtilis* and *E. coli* enzymes function as monomers while the *B. bacteriovorus* RppH appears to be a dimer (Messing et al. 2009; Piton et al. 2013). Consistent with the fact that the *B. subtilis* enzyme does not show a preference for the 5′ position of its substrate, a structure of *B. subtilis* RppH with RNA showed no density for the 5′ base but clear density for a guanosine bound far from the Nudix motif that was revealed to correspond to the second position of the bound RNA (Piton et al. 2013). Our biochemical studies showed that MERS1 also does not have a strong preference for nucleotides in the 5′ position of its motif but does show a strong preference for guanosines in the sixth, seventh, and eighth positions, suggesting the bound GTP in our MERS1–GTP structure likely does not represent the binding position of the 5′ moiety of its RNA substrate. To address this question and obtain insight into RNA binding by MERS1, we next obtained crystals of MERS1 in complex with an RNA binding site, 5′-GAGAGGGGGGUU-3′, and solved the structure to 2.6 Å resolution (Fig. 6A). The final structure has *R*_work_/*R*_free_ values of 22.0%/25.1% (Table 2).

The MERS1–RNA structure contains two MERS1 subunits in the ASU. Superimpositions show that the two MERS1 molecules have the same overall structure (superimposition of their Cα atoms resulted in an rmsd of 0.5 Å). The two MERS1 subunits in the ASU do not form significant interactions that would be suggestive of a dimer. Nor were any other possible MERS1 oligomers observed due to crystal packing, indicating that MERS1 does not form higher-order oligomers upon RNA binding. The helical interaction derived from crystal packing in the MERS1–GTP complex was also not observed in this structure, which harbors different packing. In the structure, density is evident for four RNA nucleotides bound to each MERS1 subunit (Fig 6A). The four nucleotides bound in both MERS1 molecules in the crystallographic ASU adopt the identical conformation and hence bind the same (Supplemental Fig. S3A). Weak density is present for RNA 5′ to the first constructed nucleotide, which would be close to the Nudix motif, however RNA molecules could not be unambiguously fit into this density (Supplemental Fig. S3B). This appears consistent with protein–RNA crystal structures wherein density is generally visible only for tightly bound nucleotides (Piton et al. 2013; Soufari and Mackereth 2017; Pabis et al. 2018). The electrostatic surface of the MERS1–RNA structure reveals that the RNA binding pocket is highly electropositive, indicating charge as important in nucleotide binding (Fig. 6B). Furthermore, shape complementarity appears to play a role as each base inserts into pockets within the larger binding site (Fig. 6B). Strikingly, the third nucleotide constructed in the structure, which is the best resolved of the RNA, overlaps the position of the guanine nucleotide in our MERS1–GDP structure (Fig. 6C). The phosphate moieties of the two overlap exactly while the bases are in the same location but harbor different orientations, likely because of conformational changes induced in the pocket upon binding the RNA substrate (see below). The well-resolved nature of this third guanosine suggests it as the linchpin in RNA binding and supports the notion that the GTP binding site in the MERS1–GTP structure does not represent the location of the bound 5′ nucleotide of its RNA substrate. Of the four bound RNA nucleotides in the MERS1–RNA complex, three have contacts that would provide selectivity for guanine bases and hence they were constructed as guanosines. This is consistent also with our biochemical data that revealed guanines in the center of the RNA site were key for binding by MERS1. The identity of the first observed nucleotide (5′) is not unambiguous. However, it is far enough from the active site to allow room for 3 to 4 nucleotides.

Comparison of the MERS1–RNA and MERS1–GTP structures revealed that RNA binding induces conformational changes in the RNA binding pocket (Fig. 6C); Superimposition of the MERS1–GTP and MERS1–RNA structures results in an rmsd of 1.3 Å for corresponding Cα atoms with the significant conformational changes between them found in the RNA binding pocket. Specifically, L2, comprised of residues 222–230, which covers the RNA binding pocket and is unique to MERS1, shifts by ∼3–4 Å to accommodate the RNA. The largest conformational change however occurs within residues 111–128 whereby the helix composed of residues 116–125 shifts as a unit away from the RNA pocket. Trp118, located on the helix, rotates out of a hydrophobic pocket concomitant with the transition of Gly113–Pro114–Met115 such that the proline side chain occupies the space vacated by the Trp118 side chain (Fig. 6C). These combined changes create a RNA binding site with pockets suitable for the insertion of the bases and prevent steric clash that would otherwise result.

The MERS1–RNA structure shows that there are a large number of contacts between the RNA and MERS1 (Fig. 6D). The phosphate group of the first resolved nucleotide (numbered 1) is contacted by Arg222. MERS1 residue Ser112 hydrogen bonds with its base while Trp283 makes stacking interactions to the base. The phosphate group of nucleotide 2 is contacted by Tyr197, Trp283, and Arg284. The guanine base O6 atom of nucleotide 2 hydrogen bonds to the amide nitrogen of Arg222 (Fig. 6D). The base of nucleotide 2 is also sandwiched between the side chains of Arg219, Asn221, and Met115. The side chain of Asn221 also H bonds to the sugar hydroxyl of the nucleotide. Nucleotide 3 is wedged in between the MER1 domains in the location occupied by GTP in the MERS1–GTP complex. The O6 and N1 atoms of the nucleotide 3 guanine make hydrogen bonds to the amide nitrogen and carbonyl oxygen of Gln276. The guanine base is also anchored into the pocket by hydrophobic and stacking interactions from Tyr197, Leu274, Arg284, and Val287. The ribose sugar hydroxyl group is hydrogen bonded to the side chain of Tyr289. The base for the last nucleotide has a weak density. However, its phosphate group is well resolved and contacted by Tyr289, Arg356, Tyr360, Tyr365, and Arg364. The guanine N2 atom is positioned to hydrogen bond with the carbonyl oxygen of Ile232. G-rich nucleotides, like the one bound by MERS1, are known to form higher-order structures such as G-quadruplexes (Kwok and Merrick 2017). MERS1 stabilizes its RNA in a single-stranded conformation. In fact, the RNA is deeply embedded in MERS1, raising the question of how it gains entry into this binding site. This appears to be explained by the apo MERS1 structure, which revealed that a key part of the RNA binding region is disordered in the apo state. The binding and stabilization of the single-stranded RNA may thus occur concomitant with the folding of MERS1 residues 354–380.

### Biochemical test of MERS1–RNA structure

**FIGURE 7.**
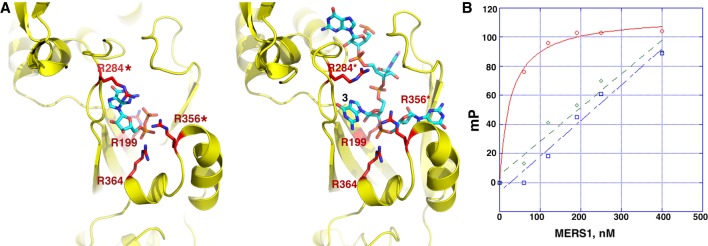
Testing structure-based RNA binding predictions. (*A*) Key arginine residues that contact the GDP (*left*) and RNA (*right*) in MERS1 complexes that were selected for mutagenesis and FP binding studies. MERS1(R284A) and MERS1(R356A) were successfully purified and used in FP binding studies in *B*. (*B*) FP binding isotherm shown in red, green and blue, respectively, of WT MERS1, MERS1(R284A) and MERS1(R356A), binding to the RNA, 5′ GAGAGGGGGUU-3′.

The MERS1–RNA structure revealed that the RNA binding pocket is particularly rich in arginine residues, which are involved in numerous contacts with the RNA. To test the roles of arginines the structure indicates as important in RNA binding, we generated R199A, R284A, R356A, and R364A substitutions (Fig. 7A). The R364A and R199A were not expressed in soluble form and hence could not be tested. However, the MERS1 R284A, and R356A mutants were soluble and could be purified to homogeneity for binding studies (Fig. 7B). The FP studies showed these mutations resulted in weak binding to the RNA substrate (Fig. 7B). Because RNA binding drives folding of the C-domain, which is important in RNA binding, the effects of these mutations may also reflect an effect of folding of this domain as well as impaired contacts between the residue and the RNA.

**FIGURE 8.**
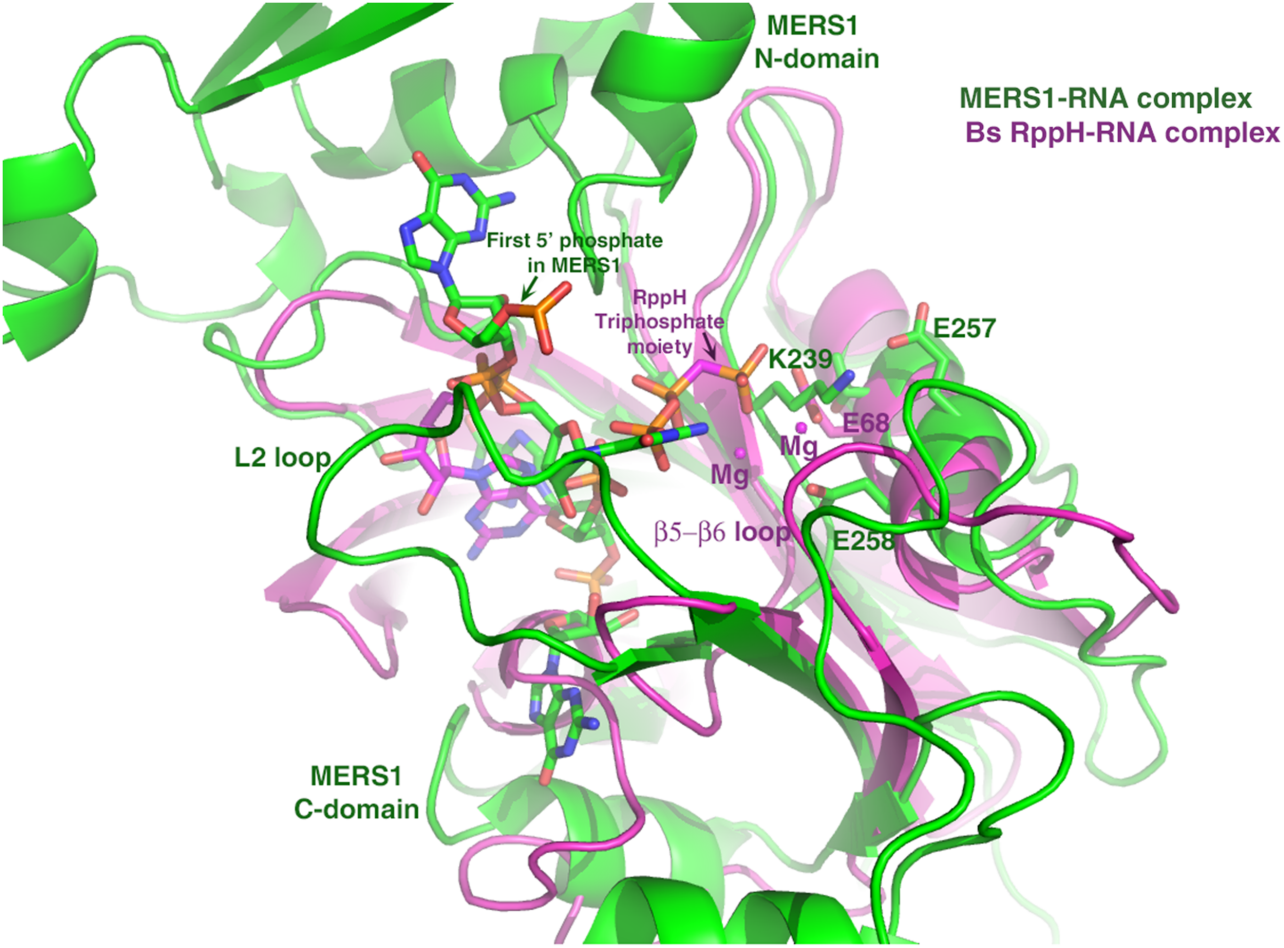
Comparison of MERS1 and *B. subtilis* RppH catalytic pocket. Superimposition of the MERS1-RNA complex (green) onto the *B. subtilis* RppH-RNA complex (magenta) (pdb code: 4JZV). The resulting rmsd is 2.5 Å for corresponding Cα atoms. In the *B. subtilis* RppH structure, the second guanine nucleotide that is specifically recognized is far from the active site. In the RppH structure, the 5′ base was not resolved, only the triphosphate group (labeled). The most 5′ nt in the MERS1 structure is also labeled. The position of the Lys239 in MERS1 clashes with a key magnesium ion in the RppH structure as well as a putative catalytic base. Either of MERS1 Nudix residues Glu257 and Glu258, are however, in position to serve as a possible catalytic base.

Our combined data suggest that MERS1 may bind its RNA substrate similar to *E. coli* and *B. subtilis* RppH proteins in which nucleotides that are located 3′ to the 5′ triphosphorylated nucleotide are key for binding specificity and stability (Piton et al. 2013; Vasilyev and Serganov 2015). MERS1, however, binds more nucleotides of its RNA substrate. This can be explained as the MERS1-RNA structure shows that residues in its N-domain and the C-terminal region that are not present in RppH proteins participate in RNA binding (Fig. 8). The question remains as to whether MERS1 uses a similar mechanism for hydrolysis as other Nudix hydrolases such as RppH, despite lacking one of the Nudix motif acidic residues. Mechanisms have been proposed for several Nudix hydrolases that involve catalytic bases either located in the Nudix motif or outside the motif on active site loops (Mildvan et al. 2005). Studies on RppH proteins suggest that the catalytic base is an acidic residue(s) in the Nudix signature sequence (Piton et al. 2013; Vasilyev and Serganov 2015). In the MERS1 Nudix motif, the most amino-terminal acidic residue is a threonine and this part of the active site in MERS1 also contains a conserved lysine, Lys239, which clashes with the positions of one of the magnesium ions observed in some RppH structures. Studies on the *E. coli* RppH suggest that the acidic residues corresponding to MERS1 Glu257, and Glu258 (Glu56 and Glu57 in *E. coli* RppH) may function in magnesium binding and catalysis (Vasilyev and Serganov 2015). In particular, Glu56 is in position to function in catalysis in the *E. coli* RppH (Vasilyev and Serganov 2015). This would be consistent with analyses showing that mutation of MERS1 residues Glu257 and Glu258 to alanines impairs RNA processing in vivo (Sement et al. 2018). However, studies have shown that it is challenging to definitively ascribe a residue as the catalytic base in Nudix hydrolases as substitution of many active site residues can either impair or abrogate binding (Piton et al. 2013; Vasilyev and Serganov 2015).

Our discovery of the fortuitously bound helix in the MERS1–GTP structure, which inserts an acidic residue close to the Nudix motif, could explain how MERS2 binding activates MERS1 activity if this residue can substitute for the missing acidic residue in the MERS1 Nudix motif and possibly contributes to magnesium binding, for example. The activities of bacterial RppH proteins and eukaryotic decapping enzymes are activated by other proteins (Charenton et al. 2016; Valkov et al. 2016, 2017). Data suggest multiples of these proteins impact activity by providing a larger binding surface for RNA interaction, and while one study suggested that the DapF protein affected the structure of *E. coli* RppH to favor its RNA binding conformation, a second study did not detect significant conformational changes in the RppH-DapF complex and led to the suggestion for a dual mechanism of stimulation that depends on the nature of the substrate whereby long RNAs, as in the eukaryotic decapping enzymes, provide a larger RNA binding surface, while for shorter RNA substrates, RppH dynamics was suggested (Gao et al. 2018; Wang et al. 2018). The interaction of the MERS2-like helix with MERS1 does not appear to mediate any structural change. Instead, it inserts an acidic residue close to the Nudix fold, which may participate in catalysis. Thus, future studies will be needed to elucidate the specific roles of MERS1 residues in catalysis and how it is activated by accessory proteins.

## MATERIALS AND METHODS

### Purification of *T. brucei* MERS1

For structural studies, the nucleotides encoding residues 37–395 of MERS1 were cloned into pET15b between the BamHI and NdeI restriction sites. *E. coli* C41(DE3) cells were transformed with the expression plasmid and protein expression was induced by addition of isopropyl β-d-1-thiogalactopyranoside (IPTG) to a final concentration of 0.5 mM for 4 h at 37°C when the cells had reached an A_600_ of 0.4–0.6. The cells were reconstituted in 25 mM Tris HCl pH 7.5, 300 mM NaCl, 5% glycerol, 0.5 mM β-mercaptoethanol and lysed with a microfluidizer. The expressed MERS1 protein was found in the soluble fraction and was purified using nickel-nitrilotriacetic acid (Ni-NTA) column chromatography. The protein was further purified by Superdex 75 size exclusion column (SEC) chromatography. MERS1 mutants were made using the Quikchange mutagenesis protocol and expressed and purified as per the WT protein.

### Crystallization and structure determination of *T. brucei* MERS1 apo and nucleotide complexes

To obtain MERS1–GTP crystals, GTP was added to the purified MERS1 protein (40 mg/mL) to a final concentration of 1 mM. Crystals were grown using the hanging drop vapor diffusion method by mixing the complex 1:1 with a reservoir consisting of 19% PEG 3350 (w/v), 0.2 M magnesium formate. The crystals grew at room temperature and took from several weeks to months to grow. Apo MERS1 crystals were generated by mixing protein at 10 mg/mL with a crystallization reagent consisting of 0.1 M [4-(2-hydroxyethyl)-1-piperazineethanesulfonic acid] (HEPES) pH 7.5 and 1.6 M MgSO_4_. The crystals grew at room temperature and appeared after several weeks. Crystals of MERS1 bound to the RNA sequence, 5′-GAGAGGGGGGUU-3′, were obtained by mixing the protein (10 mg/mL) with twofold excess RNA and then concentrating the mixture further in microcons (with 3 kDa molecular weight cutoff). The complex was concentrated threefold and then mixed 1:1 with a crystallization reservoir consisting of 0.1 M *N*-cyclohexyl-3-aminopropanesulfonic acid (CAPS) pH 10.5, 800 mM potassium phosphate dibasic, 1.2 M sodium phosphate monobasic, 0.2 M lithium sulphate. The same crystal form was also obtained by mixing the complex 1:1 with a solution of 0.1 M sodium cacodylate HCl pH 6.5, 2 M ammonium sulphate, 200 mM NaCl. The MERS1–RNA crystals took several days to 1 wk to grow. A MERS1–RNA crystal grown with the phosphate condition was ultimately used for data collection and structure determination. Data were collected for all crystals at the Advanced Light Source (ALS) beamline 8.3.1. The data were processed with MOSFLM and scaled with SCALA (Potterton et al. 2003; Leslie 2006). The MERS1–GTP crystal adopts the orthorhombic space group C222_1_, the apo form takes the hexagonal space group, P6_2_22 and MERS1–RNA crystals are monoclinic, C2. Molecular replacement (MR) using several previously solved structures with Nudix hydrolase folds were unsuccessful in solving the structure. Hence, selenomethionine (Semet) substituted protein was produced (Doublié 1997). The Semet MERS1 produced crystals with GTP under the same conditions as used to obtain WT MERS1–GTP crystals. Multiple wavelength anomalous diffraction (MAD) data were collected on one Semet crystal at beamline 8.3.1, processed with MOSFLM and scaled with SCALA (Potterton et al. 2003; Leslie 2006). The selenomethionine sites were located in AUTOSOL in Phenix resulting in a figure of merit of 0.64 (Table 1; Adams et al. 2010). AUTOSOL was used for density modification, which resulted in an excellent experimental electron density map. The structure was traced using O (Jones et al. 1991). There is one MERS1 subunit in the crystallographic ASU. The constructed structure was subjected to iterative cycles of rebuilding in O and refinement in Phenix until the *R*_free_ converged. The final model includes MERS1 residues 76–144, 156–380, 14 residues from the his-tag, 18 water molecules and one GDP molecule. The MolProbity score indicates the structure is in the top 99% of those solved to a comparable resolution (Chen et al. 2010). See Table 2 for data collection and refinement statistics.

The apo MERS1 and MERS1–RNA structures were solved by MR using the MERS1–GDP structure with the GDP and waters removed. In the apo structure, there is one subunit in the ASU and the structure was solved by MolRep. After refinement in Phenix, the structure was manually rebuilt in O (Jones et al. 1991), which revealed that several residues were disordered in this structure. After iterative rounds of Phenix refinement and manual rebuilding the structure converged to *R*_work_/*R*_free_ values of 18.8%/20.9% and the model shows excellent geometry. MolProbity analysis places the structure in the top 100% of structures solved to a similar resolution (Chen et al. 2010). The final model includes MERS1 residues 77–144, 156–353, three sulfate ions, and 234 water molecules (Table 2). The MERS1-RNA structure was solved also using MolRep and produced two solutions as there are two subunits in the ASU. After a round of refinement in Phenix, density was evident for four RNA nucleotides bound to each MERS1 molecule. Nucleotide addition reduced the *R*_free_ by 2%. After the nucleotides were added the structure was further refined and water was added. The final model includes residues 59–63, 77–145, 157–380 of one MERS1 subunit, residues 59–65, 77–144, 157–380 of the second MERS1, four nucleotides bound to each MERS1 and 41 water molecules. The structure has *R*_work_/*R*_free_ values of 22.0%/25.1%. MolProbity analysis places the structure in the top 99% of structures solved to a similar resolution (Chen et al. 2010).

### Fluorescence polarization (FP) RNA/DNA binding experiments

FP experiments were performed using a PanVera Beacon 2000 FP system at 25°C (Lundblad et al. 1996). For the experiments, purified proteins were titrated into 0.990 mL buffer composed of 25 mM Tris HCl pH 7.5, 5% (v/v) glycerol and 50 mM NaCl and containing 1 nM (final concentration) fluoresceintly labeled RNA substrate, 5′-GAGAGGGGGUU-3′, or DNA, 5′-GTGAGTACTCAC-3′. Data were fit using KaleidaGraph. Each binding curve is a representative curve from two to three technical replicates. Mutant RNA sites tested were 5′-AAGAGGGGGUU-3′ (Supplemental Fig. S2), 5′-GGGAGGGGGUU-3′, 5′-GAAAGGGGGUU-3′, 5′-GAGGGGGGGUU-3′, 5′-GAGAAGGGGUU-3′, 5-GAGAGAGGGUU-3′, 5′-GAGAGGAGGUU-3′ and 5′-GAGAGGGAGUU-3′.

### FP peptide binding experiments

In the FP peptide binding experiments, purified MERS1 was titrated into 0.990 mL buffer composed of 25 mM Tris HCl pH 7.5, 5% (v/v) glycerol and 150 mM NaCl and containing 1 nM (final concentration) fluoresceintly labeled peptide. Data were fit using KaleidaGraph. Each binding curve is a representative curve from at least three technical replicates.

### Size exclusion chromatography (SEC) analysis of *T. brucei* MERS1

For the SEC analyses of MERS1, the purified protein was concentrated to 4 mg/ml and loaded onto a Superdex 75 size exclusion column and eluted with a buffer containing 20 mM Tris HCl, pH 7.5, 150 mM NaCl, 5% (v/v) glycerol, and 1 mM DTT. The elution volume was compared to a series of protein standards to determine the molecular weights. The standards used for calculation of the standard curve are shown as blue diamonds and were cytochrome c oxidase (12.4 kDa), carbonic anhydrase (29 kDa) and albumin (66 kDa).

## DATA DEPOSITION

Coordinates and structure factor amplitudes for the MERS1 GDP-bound, apo and RNA-bound structures have been deposited with the Protein Data Bank under accession codes 6P5R, 6NL1 and 6U9X, respectively.

## SUPPLEMENTAL MATERIAL

Supplemental material is available for this article.

## Supplementary Material

Supplemental Material
