# Supplementary material for: Structures of MERS1, the 5′ processing enzyme of mitochondrial mRNAs in Trypanosoma brucei
Source: RNA. 2020 Jan;26(1):69–82. doi: 10.1261/rna.072231.119 (PMC6913127; doi:10.1261/rna.072231.119)
Supplement: Supplemental Material [file supp_26_1_69__index.html]

Structures of MERS1, the 5′ processing enzyme of mitochondrial mRNAs in Trypanosoma brucei — Supplemental Material 

# Structures of MERS1, the 5′ processing enzyme of mitochondrial mRNAs in *Trypanosoma brucei*

## Supplemental Material

- Supplemental\_Figures\_S1-S3.docx
