# Supplementary material for: Structures of MERS1, the 5′ processing enzyme of mitochondrial mRNAs in Trypanosoma brucei
Source: RNA. 2020 Jan;26(1):69–82. doi: 10.1261/rna.072231.119 (PMC6913127; doi:10.1261/rna.072231.119)
Supplement: Supplemental Material [file supp_072231.119_Supplemental_Figures_S1-S3.docx]

Figure S1


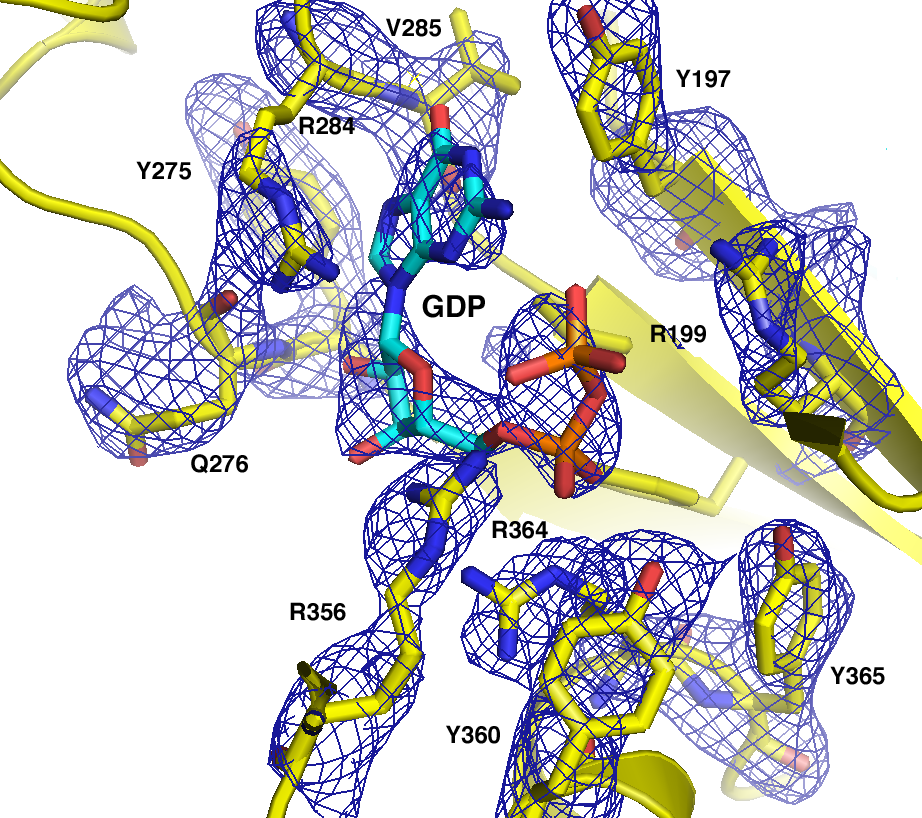


**Figure S1**. Omit map of GDP (GTP) and binding residues in the MERS1-GTP structure. The GDP and GDP-contacting residues of MERS1 were omitted and refinement run for 15 cycles. The Blue map represents Fo-Fc density contoured at 2.6 σ.

e MERS1-GTP structure. The GDP and GDP-contacting residues of MERS1 were omitted and refinement run for 15 cycles. The Blue map represents Fo-Fc density contoured at 2.6 σ.

Figure S2


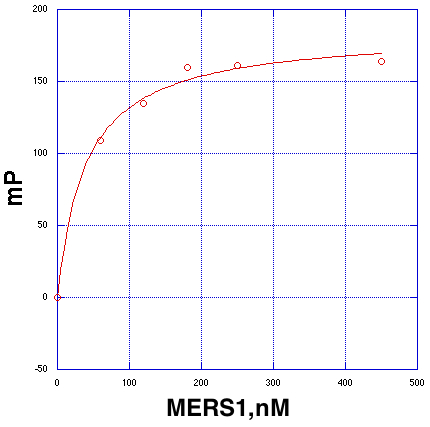


**Figure S2**. Fluorescence polarization binding isotherm of MERS1 binding to 5-fluoresceinated RNA, 5’-AAGAGGGGGUU-3’. The Y-axis is millipolarization units (mP) and the X-raxis is the protein concentration in nM. Each binding curve is a representative analysis from at least 3 technical replicates. The standard error was determined from the binding affinities of the three experiments. The resultant K_d_ was 39 ± 4 nM.

Figure S3


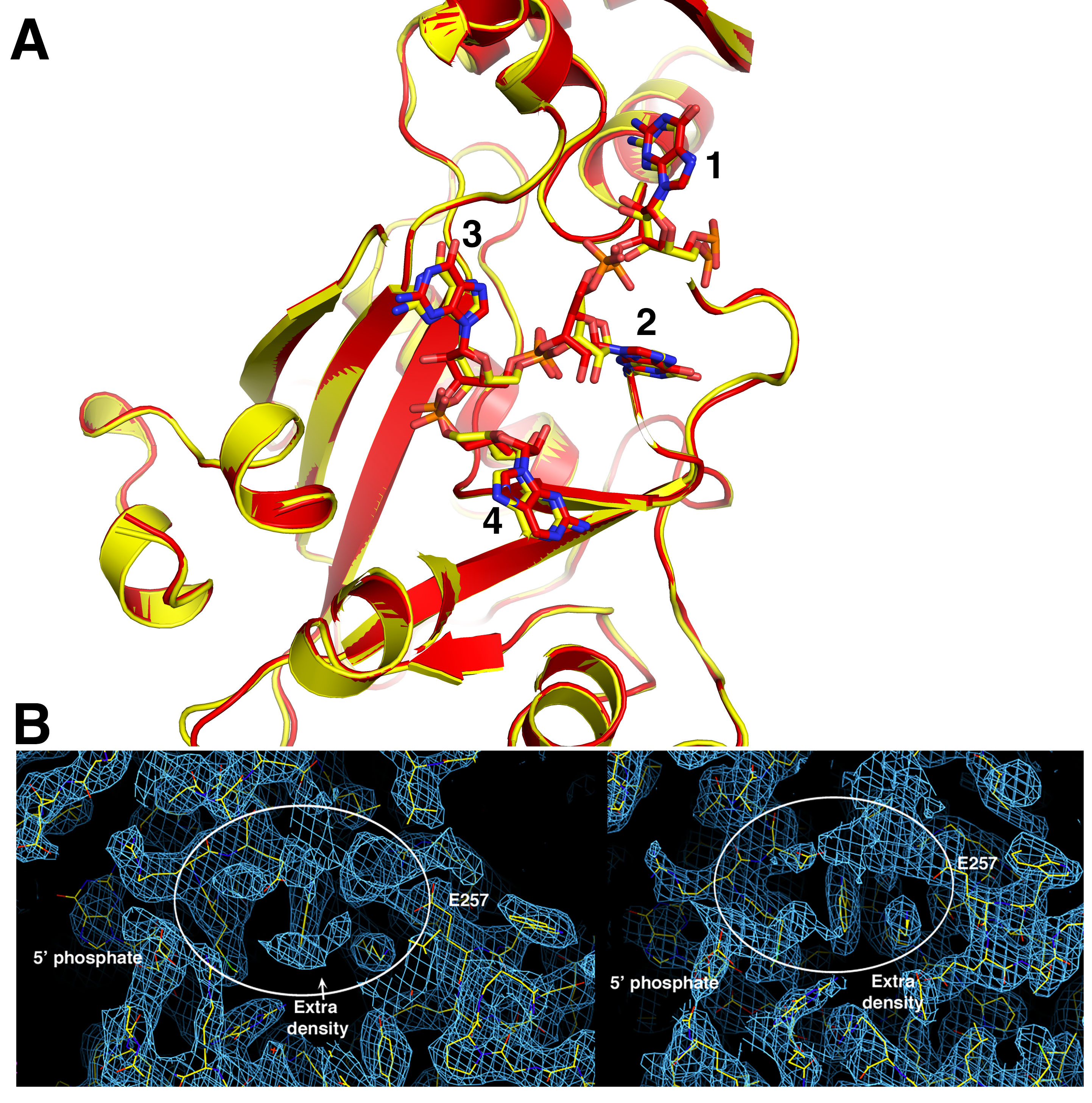


**Figure S3**. RNA binds in the same conformation for the two MERS1 subunits in the ASU. **A.** Superimposition of the two MERS1 subunits in the ASU showing that the RNA molecules bind in essentially the same conformation in each subunit. **B.** Composite omit map calculated for the MERS1-RNA structure showing that density is present between the first observed 5´ phosphate of nucleotide 1 (labeled) and Glu257 located on the MERS1 Nudix motif. Shown is electron density (blue mesh) contoured at 0.9 σ to 2.6 Å resolution for both subunits in the crystallographic ASU. The extra density is circled. Although it was present at above noise level it could not be unambiguously traced.
